# µ-Raman and GC-MS to interrogate the heterogeneity of deuterium-label incorporation on the single cell vs. fatty acid isotopologue level

**DOI:** 10.1007/s00216-026-06452-y

**Published:** 2026-04-29

**Authors:** Kara Müller, Natalia P. Ivleva, Claudia Huber, Wolfgang Eisenreich, Martin Elsner

**Affiliations:** 1 Institute of Water Chemistry, Chair of Analytical Chemistry and Water Chemistry, TUM School of Natural Sciences, Technical University of Munich, Lichtenbergstr. 4, Garching, 85748 Germany; 2 Structural Membrane Biochemistry, Bavarian NMR Center, TUM School of Natural Sciences, Technical University of Munich, Lichtenbergstr. 4, Garching, 85748 Germany

**Keywords:** Stable isotope probing, Hydrogen isotopes, Raman microspectroscopy, FAME-GC-MS, Bacteria, Metabolic activity

## Abstract

**Abstract:**

Understanding microbial metabolic functions at both bulk biomolecular and single-cell biomass levels requires precise tracing of isotope incorporation. Despite the established nature of compound-specific and single-cell stable isotope probing (SIP) approaches, a systematic comparison remains lacking. This study presents a comparative analysis of two SIP methods, utilizing deuterium from labelled glucose and heavy water to track incorporation into microbial fatty acids through gas chromatography-mass spectrometry (FAME-GC-MS) and into single cells via stable isotope Raman microspectroscopy (SIRM). Both methods exhibited consistent trends in deuterium incorporation across bacterial samples of *Sphingomonas koreensis* grown on various (deuterated) substrates in the presence of differing concentrations of D$_{2}$O. The deuteration levels of single cells and fatty acids supported one another quantitatively. Besides demonstrating good agreement between both datasets, they were also shown to synergistically complement each other. Information on different cell generations was derived from clusters of varying degrees of cell deuteration, and heterogeneities between different fatty acids and their isotopologues were revealed. Additionally, variations in carbon and deuterium sources influenced fatty acid profiles and biomass deuterations. This proof-of-principle study underscores the consistent and complementary nature of lipid-SIP and single-cell-SIP approaches, providing a benchmark for future research on microbial community dynamics and organic substrate biodegradation.

**Graphical abstract:**

**Figure:**
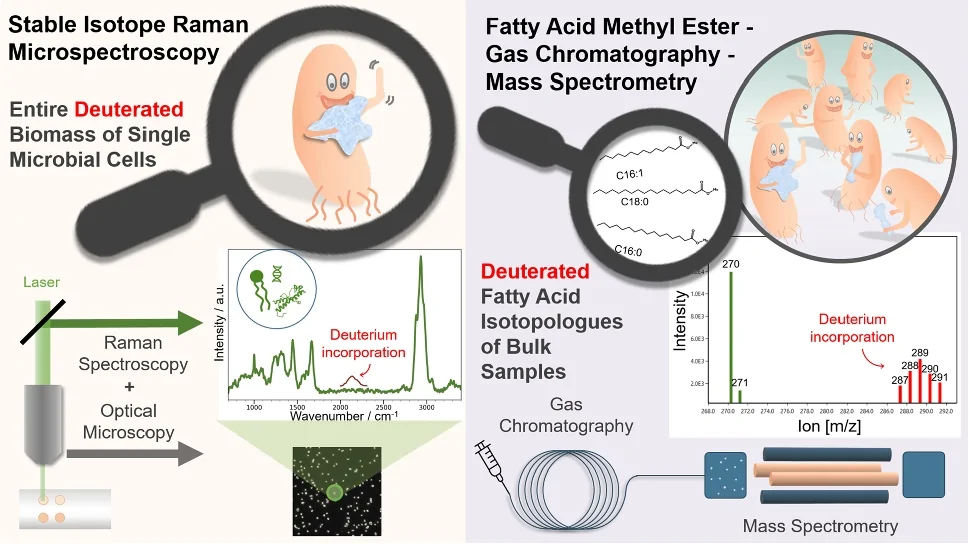

**Supplementary Information:**

The online version contains supplementary material available at https://doi.org/10.1007/s00216-026-06452-y.

## Introduction

Stable isotope probing (SIP) tracks the incorporation of isotopically labelled substrates (e.g., $^{13}$C or D) into microbial biomass or biomolecules to characterize microbial metabolic activity and function, thereby providing insights into microbial physiology and ecological roles [1–5]. Where possible, these measurements can be linked to microbial identity. In different SIP approaches, distinct biomolecular markers are targeted to trace isotope incorporation from the substrate into different biomolecules. While in nucleic acid-SIP, detection of the label in the DNA or RNA can trace the identity of degrading microorganisms [6–9], in protein-SIP, proteins are analyzed to trace the label in putative enzymes to obtain functional evidence of metabolic activity [10–14].

By focusing on membrane lipids, lipid-SIP targets biomolecules that can contain phylogenetic information and provide insights into microbial physiologic states. Compared to nucleic acid-based approaches, lipids are generally easier to extract and analyze and require lower isotopic enrichment. Isotope ratios of fatty acids are typically quantified by mass spectrometry following the extraction of specific lipid fractions, such as phospholipid fatty acids (PLFAs) or total fatty acids. Although it is less specific to individual microorganisms compared to nucleic acid-SIP, it is well-suited for studies of complex environmental samples with a limited number of active organisms, or for analyzing microorganisms that possess specific biomarker fatty acids, such as methanotrophs.

$^{13}$C labels have been widely used in lipid-SIP studies, since they enable direct interrogation of central carbon metabolisms [15]. Prominent applications include resolving microbial contributions to chemoautotrophic and methane-based primary production, including methane oxidation-coupled autotrophy across a broad range of environments [16–21]. In addition, lipid-SIP has been used to trace microbial assimilation of plant-derived carbon [22, 23] and of anthropogenic carbon sources including organic pollutants such as herbicides [24], hydrocarbons [25–27], and (micro)plastics [28–31]. On the other hand, deuterium labels offer broader substrate availability, lower cost, and a much lower natural isotopic background. General microbial activity can be measured by applying D$_{2}$O, which can be combined with tracing inorganic or organic $^{13}$C labels into fatty acids [32–39]. Moreover, Alexandrino et al. used styrene-d$_{8}$ to identify active styrene degraders in biofilter samples, demonstrating that also deuterated carbon substrates can serve as a labelling source for lipid-SIP studies [40]. In other studies, the degradation of hexane-d$_{14}$ in biofilters [41] and of pristane-d$_{40}$ in sediment microcosms [42] have been analyzed with lipid-SIP.

In addition to SIP approaches targeting distinct biomolecular markers, there are also biomass-SIP methods that investigate the label uptake into bulk biomass or single microbial cells [4, 43, 44]. The most common single-cell-SIP (SC-SIP) methods are nanoscale secondary ion mass spectrometry (nanoSIMS) and stable isotope Raman microspectroscopy (SIRM), which can resolve heterogeneities within microbial communities. NanoSIMS has a high mass resolution and spatial precision down to 50 nm and can be applied for isotopes of various elements in parallel (e.g., H, C, N, and P). This method has been applied to study different biogeochemical processes (see [45] for a review). However, NanoSIMS only works for dry samples, requires laborious sample preparation, and is cost-intensive. In comparison, SIRM is a non-destructive method with much simpler and faster sample preparation and measurements, which also works with aqueous samples and has a spatial resolution down to 1 µm, sufficient to resolve single microbial cells. Drawbacks are interferences in autofluorescent samples and the lack of exact information on chemical composition, since the vibrational spectra only provide chemical fingerprints (see [46, 47] for reviews on SIRM). Most commonly, $^{13}$C labels are used in combination with Raman microspectroscopy to trace carbon from organic [48–58] or inorganic [59–61] substrates directly or via reverse labeling into single microbial cells. Moreover, D labels are applied in the form of D$_{2}$O to measure general metabolic activity, i.e., in response to antibiotic treatment [62–68]. To study specific metabolisms, D$_{2}$O can be used in combination with non-labelled carbon substrates [69, 70]. Alternatively, deuterated carbon substrates are applied (e.g., glucose and naphthalene [51, 69, 71, 72], fatty acids [73–75], or (micro)plastics [76]). Common applications of SC-SIP approaches are to study cellular metabolisms including microbial dormancy and microbial activity under different conditions, to reveal phenotypic heterogeneity in the context of nutrient limitations or environmental fluctuations, and to investigate symbiotic interactions and cross-feeding in microbial communities [4]. While SC-SIP methods provide no taxonomic information, they can be combined with other methods such as fluorescence in situ hybridization (FISH) to create a link between phenotype and genotype or microfluidics for cell-sorting and subsequent genomic analyses [49, 54, 60, 62, 63, 77–79].

Tracing a stable isotope label from a substrate into specific metabolites (i.e., fatty acids) and into individual cells of the same population allows testing the robustness and consistency of the respective methods without the need for calibration. Moreover, potential differences between the resulting data sets could highlight the complementarity of approaches operating at different analytical scales, ranging from bulk measurements and single metabolites to individual cells and total biomass. Yet, a systematic comparison of these approaches at different analytical scales is still lacking.

Although Stiebing et al. previously combined SIRM with fatty acid methyl ester gas chromatography (FAME-GC), their study addressed a different research question [73]. Their focus was on demonstrating the complementary value of spatially resolved Raman imaging and bulk FAME-GC for monitoring fatty acid distributions. To this end, they traced the uptake and storage of deuterated exogenous fatty acids in macrophage cells, rather than investigating substrate utilization and metabolic activity.

Another study in which lipid-SIP was combined with vibrational spectroscopy was published by Kukal et al. [80]. They traced deuterium from glucose-d$_{7}$ and D$_{2}$O into triacyl glycerides in yeast samples and bulk biomass, rather than single cells. Both infrared (IR) and Raman spectroscopy were used to determine biomass deuteration, showing similar qualitative trends. However, the absolute values differed significantly: the ratio of the areas under the C-H and C-D stretching bands, indicative of biomass deuteration degrees, ranged from 0.5 ± 0.1 for IR to 3.03 ± 1.09 for Raman measurements of the same sample. This highlights the need for a quantitative cross-referencing of spectroscopic biomass data with FAME-GC-MS data, which was not provided in their publication. Here, we therefore aim to take a step further by quantifying D label incorporation from different sources into single bacterial cells based on ratios of different Raman bands, and into fatty acids based on single ion traces. This approach allows overlapping fatty acid peaks in chromatograms to be distinguished and isotopologue distributions to be derived for a quantitative comparison with single-cell Raman data.

The main objective of our present study was to evaluate whether combining two well-established, orthogonal SIP techniques—SIRM and FAME-GC-MS—into a single, cohesive approach provides mutual validation of deuteration levels and fractions, and to assess its synergistic value. Specifically, we examined the bacterial physiology of *Sphingomonas koreensis* by analyzing heterogeneities in the Raman data to determine whether information on active and non-active cells, as well as cell generation numbers, could be obtained. We also compared the labelling patterns of FAMEs and analyzed their deuterated isotopologues. Additionally, we investigated how bacterial activity and the cellular fatty acid profiles change in response to different carbon substrates (glucose or lactate) and deuterium sources (D$_{2}$O or glucose-d$_{12}$) using both methods.

## Materials and methods

### Incubation of *Sphingomonas koreensis* with different non-deuterated/deuterated substrates

Unless otherwise stated, all chemicals and materials were purchased from Carl Roth (Karlsruhe, Germany) and Sigma-Aldrich (Steinheim, Germany) and autoclaved for incubation experiments at 120 $^{\circ }$C. All incubation and sample preparation steps during which vials were opened were performed in a laminar flow hood (Stella Air, 4 more Labor, Zefa Group, Harthausen, Germany) to prevent microbial contamination.

The used bacterium strain was previously isolated from an aged polylactic acid suspension in water [48] and 16S rRNA gene sequencing (SepsiTest™ Blast, Molzym) revealed 98.9% sequence identity to *Sphingomonas koreensis*, indicating close phylogenetic relatedness. Although this level of identity does not allow unequivocal species-level assignment, the isolate is hereafter referred to as *S. koreensis* for simplicity. For all experiments (see Table 1), the cells were grown in an optimized minimal medium (MM) with the same salt concentrations as in [48, 76], supplemented with either D-glucose, D-glucose-d$_{12}$ (97 atom% D, Santa Cruz Biotechnology, USA), or sodium L-lactate and 0%, 80%, or 96% D$_{2}$O (99.9 atom % D). To ensure that the bacteria were already adapted to either D-glucose or L-lactate as carbon sources, they were pre-incubated in MM with the corresponding substrate for 3 days.

**Table 1:** The experiments consisted of the following combinations of carbon substrates and deuterium sources

Carbon substrate	D$_{2}$O content
D-glucose	0%
D-glucose	80%
D-glucose-d$_{12}$	0%
D-glucose-d$_{12}$	96% $^{1}$
Sodium L-lactate	0%
Sodium L-lactate	80%

$^{1}$Maximum deuterium content was chosen; 100% D$_{2}$O was experimentally impractical

To achieve consistent D$_{2}$O concentrations across media with different carbon sources, media without D$_{2}$O, containing 80% D$_{2}$O or 96% D$_{2}$O, were first prepared without any carbon source, then divided into 5 mL aliquots in 15-mL conical plastic centrifuge tubes for the various experiments. Afterwards, 110 µL of 1 mol/L stock solutions of D-glucose, D-glucose-d$_{12}$ (97 atom % D, Santa Cruz Biotechnology, USA), or sodium L-lactate were added to the respective subsamples. The pre-incubated bacteria suspensions, grown on either glucose or lactate, were washed (centrifuged at 4500 rpm and 4 $^{\circ }$C for 10 min, supernatant removed, and cell pellet resuspended) in 5 mL organic carbon-free MM to remove non-deuterated C substrates. The obtained organic carbon-free cell suspensions were split into fractions for the experiments with different D sources and ratios (see Table 1) and washed again with MM of the according D$_{2}$O ratio. Aliquots of 200 µL of cell suspensions were then added to each experiment (samples comprising 5 mL of MM with different D$_{2}$O ratios plus 110 µL of carbon source solutions). After thorough mixing, 150 µL of each sample was pipetted in triplicate into a sterile microplate to determine optical density at 600 nm (OD600) values using a BioTek Synergy HT microplate reader (Agilent Technologies, USA). OD600 values ranged from 0.05 to 0.07 after blank subtraction. The samples were incubated in the 15-mL centrifuge tubes, enclosed with a lid, in an incubator shaker at 32 $^{\circ }$C and 120 rpm. After 4 days, all samples were ventilated (soft shaking of opened tubes in a laminar flow hood), and significant growth was already optically observed. After 6 days, samples for OD600, SIRM, and GC-MS measurements were taken.

### Stable isotope Raman microspectroscopy measurements

After OD600 measurements were performed in a sterile microplate (according to the same procedure as described above), the 150 µL subsamples were recombined and prepared for SIRM measurements. To this end, cells were pelleted in a centrifuge at 4500 rpm and 4 $^{\circ }$C for 3 min. The cell pellets were washed two times in 800 µL MilliQ water and finally resuspended in 500 µL MilliQ water. Three 1 µL droplets of 1:100 dilutions in MilliQ water of each sample were spotted on an aluminum-coated glass slide (EMF Dynasil, USA) and allowed to dry at room temperature. Raman spectra were acquired using a green frequency-doubled Nd:YAG laser with a wavelength of 532 nm on a WITEC alpha300 apyron automated confocal Raman microscope (Oxford Instruments, Ulm, Germany). The measurements were automated, using the WITec software ParticleScout with the following parameters: 8 mW at the sample, maximal 15 accumulations of 0.5 s integration time in the mode “Optimize fast” with a low signal of 50, a signal-to-noise ratio of 110, and a spectral autofocus ranging from −3 to 3 (0.05, 1). The instrument was equipped with a 300 g mm$^{-1}$ diffraction grating and a 100× objective (Zeiss EC Epiplan-Neofluar, numerical aperture, NA = 0.9, working distance, WD = 1 mm, Zeiss, Germany).

### Spectra processing and calculation of deuterations

The Python scripts used to process the spectra and to calculate deuteration degrees and fractions were written by the authors with the use of the AI language model ChatGPT (OpenAI) for a few minor code snippets and clarifications regarding data structures. All code was independently verified, tested, and integrated by the authors, who take responsibility for its correctness and functionality. A detailed structure of the code is appended in the supporting information (SI). In short, the pre-processing of single-cell Raman spectra consisted of a wavenumber correction based on daily Si-wafer measurements, smoothing with a Savitzky-Golay filter, baseline correction with a statistics-sensitive non-linear iterative peak-clipping (SNIP) algorithm, and a normalization to the integral of the C-D and C-H stretching vibrations. Band areas of C-D and C-H stretching vibrations were calculated as the sum of five Gaussians that were fit to the spectra, and the ratio of the C-D / (C-D + C-H) band areas was used to describe single-cell deuteration degrees.

**Fig. 1:**
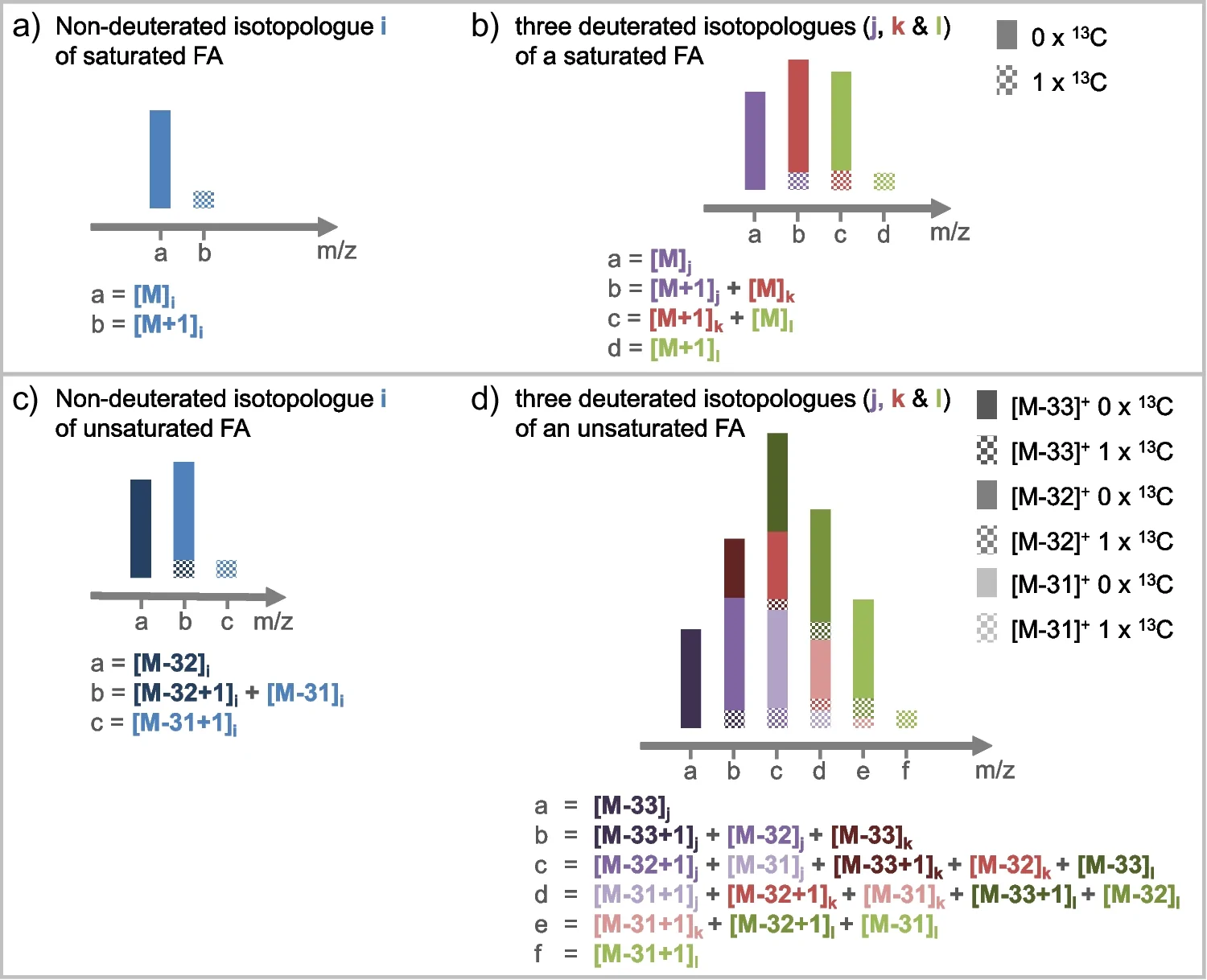
Scheme of the model used to describe the mass spectra of **a** non-deuterated saturated, **b** deuterated saturated, **c** non-deuterated unsaturated, and **d** deuterated unsaturated FAMEs. Three D-isotopologues are schematically shown for deuterated FAMEs (blue, red, and green), although up to 14 isotopologues have been observed in our experiments for one fatty acid

### Fatty acid methyl ester generation and extraction

The residual 4.1 mL of the samples were used for FAME-GC-MS analyses. To this end, cells were pelleted in a centrifuge at 4500 rpm and 4 $^{\circ }$C for 10 min. The supernatants were discarded, and the cell pellets were transferred to 1.5-mL glass vials with screw caps and crushed with a spatula to increase the surface area. They were then freeze-dried and stored at −27 $^{\circ }$C until usage, resulting in cell pellets of 0.7 to 2.7 mg. For the methylation of the fatty acids to their methy lesters, 500 µL of HCl in methanol (3 M, derivatization grade) was cold-pipetted to each freeze-dried cell pellet. The samples were gently shaken to suspend the cell pellet in the methanol and shortly opened afterwards to balance the increased pressure. The derivatization took place in a ventilated oven connected to an exhaust system at 85 $^{\circ }$C for 2 h. Note that during the derivatisation, the pressure increased which can lead to broken lids. Therefore, the lids were not tightened fully, and evaporation losses were balanced by adding more HCl in methanol. After derivatisation, the samples were dried under a gentle nitrogen flow at room temperature. FAMEs were extracted in 100 µL dry hexane. After thorough mixing, 80 µL per sample was transferred for measurements into glass GC vials.

### Gas chromatography—mass spectrometry measurements

The FAME analysis was conducted using an Agilent 7890A GC system equipped with a 5979C Triple-Axis Detector MS (Agilent Technologies, USA). For each sample, three 10 µL injections were performed with a PAL autosampler (CTC Analytics AG, Switzerland) in separate runs, resulting in triplicate measurements per sample. The injector was operated in split mode with a split ratio of 5:1 and a split flow of 5.5 mL min$^{-1}$ and was maintained at a temperature of 260 $^{\circ }$C with a pressure of 1.0 bar, a total flow of 9.6 mL min$^{-1}$, and a septum purge flow of 3 mL min$^{-1}$. Separation of the target analytes was performed on an Rxi^®^ 5ms column (30 m length, 0.25 mm inner diameter, and 0.25 µm film thickness; Restek, USA) with a constant helium flow rate of 1.1 mL min$^{-1}$. The column was protected by a pre- and post-column (deactivated fused silica, 0.25 mm inner diameter; Agilent Technologies, USA), with the post-column temperature maintained at 250 $^{\circ }$C throughout the analysis. The GC oven was programmed as follows: an initial hold at 150 $^{\circ }$C for 3 min, followed by a ramp of 3 $^{\circ }$C min$^{-1}$ to 231 $^{\circ }$C (hold for 1 min) and a subsequent ramp of 15 $^{\circ }$C min$^{-1}$ to 300 $^{\circ }$C. Ionization was performed using a high-efficiency source (HES) at 70 eV, with the ion source temperature set to 230 $^{\circ }$C. The quadrupole temperature was set to 150 $^{\circ }$C. Three full-scan spectra (mass range 200–350) were recorded for each sample after a 5-min solvent delay.

### GC-MS data analysis

Only the methyl esters of the most abundant C$_{16}$ and C$_{18}$ fatty acids were included in this study, specifically C$_{16:0}$, C$_{16:1}$, C$_{18:0}$, C$_{18:0}$-OH, and two C$_{18:1}$ isomers (designated C$_{18:1a}$ and C$_{18:1b}$) with different retention times.. These were previously identified from full-scan mass spectra, which featured the FAME-specific McLafferty-ion at m/z = 74. For the detailed isotopologue analysis, we focused on the [M]$^{+}$ ion for saturated FAMEs and the [M-31]$^{+}$, [M-32]$^{+}$, and, in the case of deuteration, [M-33]$^{+}$ ions for unsaturated FAMEs (see SI, Fig. S1, for structural formulas of the fatty acids and the considered fragments). Additionally, all isotopologues containing one $^{13}$C atom were included in the analysis (see Fig. 1).

**Fig. 2:**
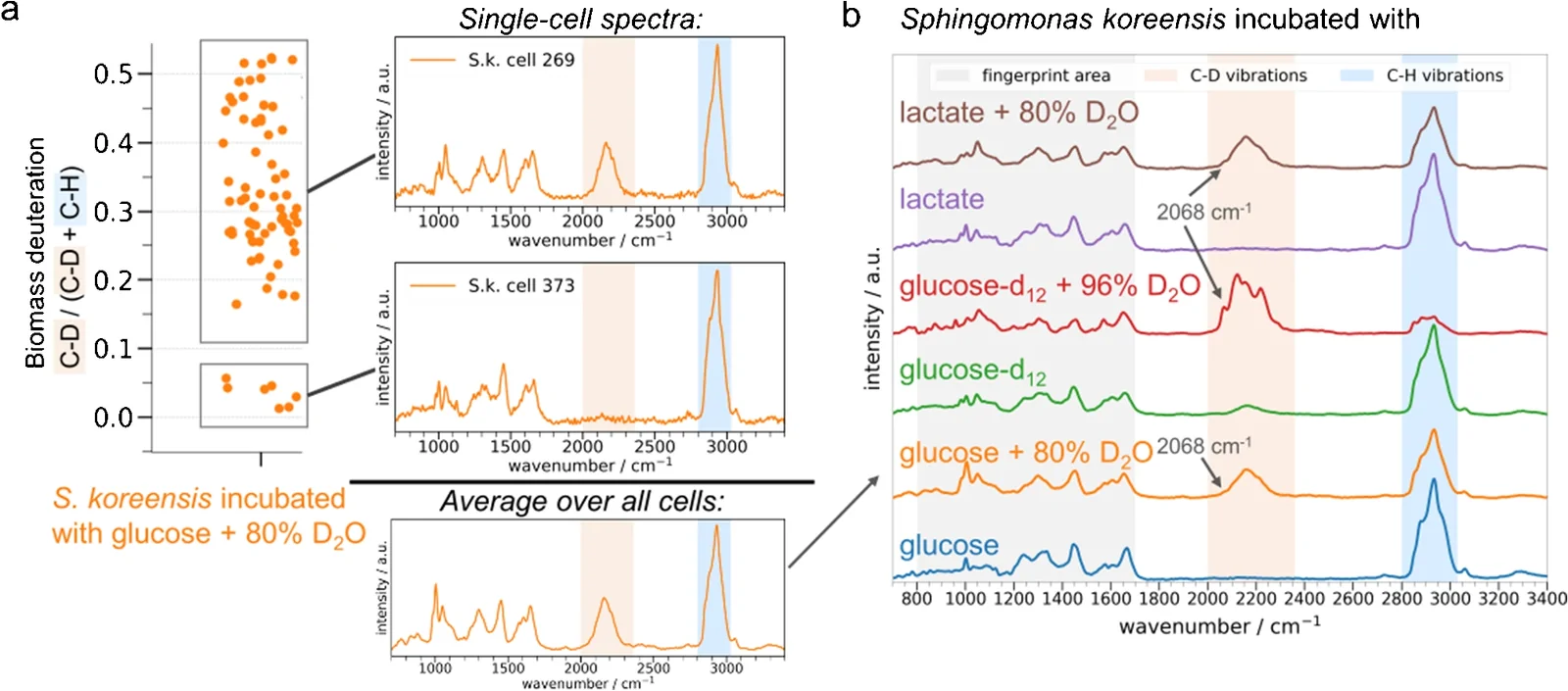
(a) Biomass deuterations are calculated as the ratio of the peak area of the C-D Raman band over the sum of the C-D and C-H bands for each single-cell Raman spectrum. The mean spectrum is the average over all cells, including non- and deuterated ones. (b) Mean Raman spectra of n single *S. koreensis* cells incubated with the following substrates: glucose, *n* = 47; glucose with 80% D$_{2}$O, *n* = 75; glucose-d$_{12}$, *n* = 98; glucose-d$_{12}$ with 96% D$_{2}$O, *n* = 101; lactate, *n* = 57; and lactate with 80% D$_{2}$O, *n* = 91. The Raman spectra were normalized to the sum of the areas of the C-D (1960–2400 cm$^{-1}$) and C-H (2800–3030 cm$^{-1}$) regions

The data obtained with the Agilent GC-MS system was converted to .mzML files using the software OpenChrome (Lablicate, Germany) and then to .txt files using the MSExportGUI [81] of ProteoWizard. Data analysis and visualization were then performed using a Python script developed by the authors. For select small sections of code, the AI language model ChatGPT (OpenAI) was consulted. The authors verified, tested, and incorporated all code themselves and take responsibility for its accuracy and functionality. A detailed description of the data processing and analysis workflow is provided in the SI. In short, trapezoidal integrals in specific retention time ranges were calculated for the single mass traces of the corresponding fragment ions to determine the abundances of non-deuterated FAMEs and for the manually determined mass traces of interest for deuterated FAMEs. Linear least-squares problems with bound constraints were constructed as described in the SI and solved using the lsq_linear algorithm implemented in SciPy to deconvolute the contributions of the different D-isotopologues.

## Results and discussion

### Biomass deuterations as calculated from Raman spectra

In Raman microspectroscopy, the laser beam is focused onto one single cell, and vibrations of all biomolecules within the cell are excited, resulting in whole-organism spectra. This results in numerous overlapping Raman bands. Raman spectra of microorganisms can be divided into the fingerprint region (up to 1700 cm$^{-1}$), C-H vibrations (2800–3100 cm$^{-1}$), and the Raman-silent region in between (Fig. 2a, lower single-cell spectrum). Frequencies of stretching vibrations are inversely proportional to the square root of the reduced mass of the vibrating atoms. Therefore, the high relative mass difference of D vs. H leads to a significant shift of C-H stretching vibrations into the normally Raman-silent region. Consequently, red-shifted C-D vibrations in the frequency range of 2000–2350 cm$^{-1}$ (red background) can be observed for deuterated cells (see Fig. 2a, upper single-cell spectrum).

Single-cell deuteration degrees are commonly calculated by the band area ratios of the C-D/(C-D + C-H) stretching vibrations. An example of the distribution of the deuteration degrees of the cells, incubated with glucose and 80% D$_{2}$O, is shown in Fig. 2a, whereas the distribution data of all other samples are later discussed in Fig. 4. Due to noise and elevated backgrounds, metabolically inactive cells (see the lower box of data points in Fig. 2a) may show nominal deuterations of up to 6% as illustrated for cell 373 in Fig. 2a. Metabolically active cells (see the upper box of data points in Fig. 2a) show significantly stronger C-D Raman bands of different intensities. Figure 2b displays the mean spectra of representative cell numbers for samples cultivated with different substrates (lactate or (deuterated) glucose) and different D$_{2}$O contents as a superposition of all single-cell spectra, i.e., the averaged spectra of 47–57 *S. koreensis* cells per incubation without a deuterium source and of 75–101 cells per incubation with different D sources. These spectra indicate that deuterium was assimilated by all populations exposed to a deuterium source, with the least D incorporation in the sample with glucose-d$_{12}$ and the greatest with the combination of glucose-d$_{12}$ and D$_{2}$O.

### Fatty acid retention times vary for D-isotopologues

**Fig. 3:**
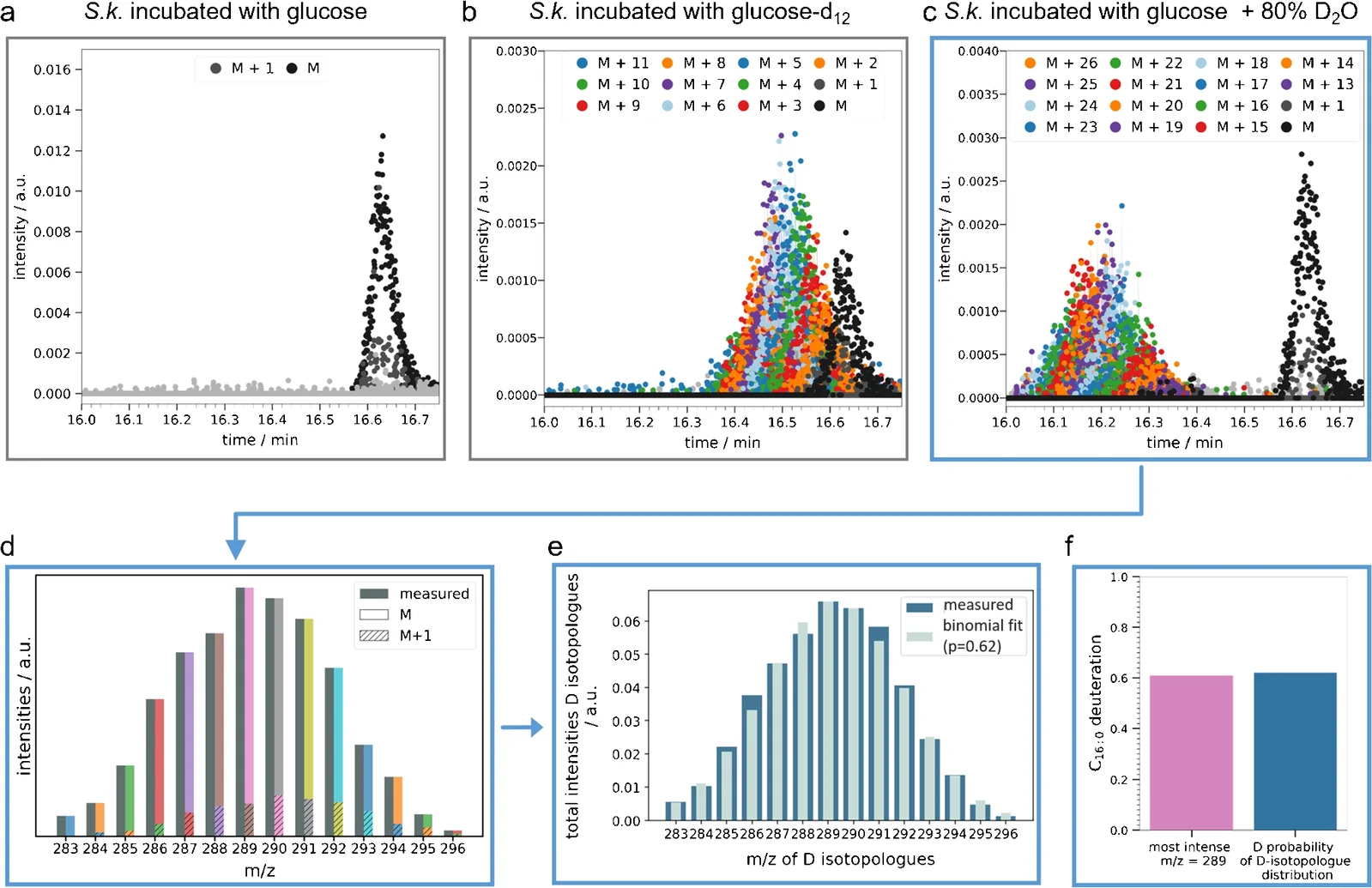
(a–c) The chromatogram sections displaying the isotopologues of the C$_{16:0}$ FAME in the range of 16.00 to 16.75 min are shown for the samples where *S. koreensis* was incubated with (a) glucose, (b) glucose-d$_{12}$, and (c) 80% D$_{2}$O. The single ion traces m/z = 270–303 are shown to cover all possible isotopologues of C$_{16:0}$. The single ion traces of interest are highlighted in different colors. (d) Based on the data from panel c, the mass spectrum of the deuterated C$_{16:0}$ FAME isotopologues is constructed. The measured intensities in grey are overlain by the deconvoluted D-isotopologue intensities resulting from the calculated contributions of the [M]$^{+}$ (colored) and [M+1]$^{+}$ ions (1 × $^{13}$C, colored with stripes). (e) A binomial distribution is fit to the D-isotopologue distribution obtained from panel d. (f) The deuteration probability of the binomial distribution in panel E is compared with the deuteration degree of the m/z with the strongest intensity (m/z = 289, 61% D), shown in panel d

The methyl esters of different fatty acids (FAMEs) within one sample were chromatographically separated based on their retention times. A representation of the peaks belonging to the non-deuterated and deuterated analogues of the C$_{16:0}$ FAME of three samples is shown in Fig. 3a–c. The peak at 16.64 min, assigned to non-deuterated C$_{16:0}$, was present in all three samples. Additional broad peaks appeared in the chromatograms of samples with a D source (Fig. 3b, c) at shorter retention times. These peaks can be assigned to deuterated analogues based on mass spectrometric information, where the retention times were found to correlate with the varying degrees of deuteration. The underlying phenomenon is that C-D bonds are shorter than C-H bonds, and physical properties are changed, such as van der Waals forces, leading to a higher vapor pressure and an earlier elution of isotopologues with more C-D bonds [82]. This is reflected by the single mass traces shown in Fig. 3b/c, which represent the chromatographic peaks of deuterated C$_{16:0}$. The deconvolution of these different deuterated isotopologues, resolved by their mass traces, is the key to assessing the heterogeneity of label incorporation in fatty acid profiles, as described in the following paragraph.

### Deconvolution of D-isotopologues

For the discussion of FAME deuteration degrees, we related the measured m/z values to isotopologues of different deuteration degrees (“D-isotopologues”). Each D-isotopologue of molecular mass M can create different ions by fragmentation. We considered the following diagnostic ions: [M]$^{+}$ for saturated FAMEs; [M-31]$^{+}$, [M-32]$^{+}$ and in the case of deuteration [M-33]$^{+}$ for unsaturated FAMEs (fragments after the elimination of a methoxyl group, a methoxyl group plus a hydrogen atom, and a methoxyl group plus a deuterium atom, respectively); and [M-113]$^{+}$ for C$_{18:0}$-OH (fragment after elimination of $C_{8}H_{17}$, see SI, Fig. S1). Additionally, each ion occurs with one $^{13}$C atom based on the natural occurrence of $^{13}$C (“$^{13}$C-isotopologue”). For each D-isotopologue, we therefore added up the intensities of ions with the same degree of deuteration, including those with (hatched bars in Fig. 3d) and without one $^{13}$C atom (plain bars of the same color in Fig. 3d). For example, if we look at the mass 289 in the chromatographic peak of C$_{16:0}$ (see Fig. 3d), it is composed of the D-isotopologue with (289–270 = ) 19 D atoms and no $^{13}$C atoms (plain pink bar) plus the D-isotopologue with 18 D atoms and one $^{13}$C atom (hatched brown bar). To calculate the abundance of the D-isotopologue with 19 D atoms, we hence summed up the share of the masses 289 that belongs to the D-isotopologue without $^{13}$C atom (M; plain pink bar) and 290 that belongs to the D-isotopologue with one $^{13}$C atom (M+1; hatched pink bar). The self-written algorithm to deconvolve the two contributions, as illustrated in Fig. 3d, is further explained in the Materials and Methods section.

When the estimated intensities of these two ions are summed up for the saturated C$_{16:0}$ FAME, a D-isotopologue distribution shown in Fig. 3e is derived. It resembles a binomial distribution with a deuteration probability of 62% for each of the 31 H atoms in the C$_{16:0}$ FAME that belong to the original fatty acid before derivatization.

**Fig. 4:**
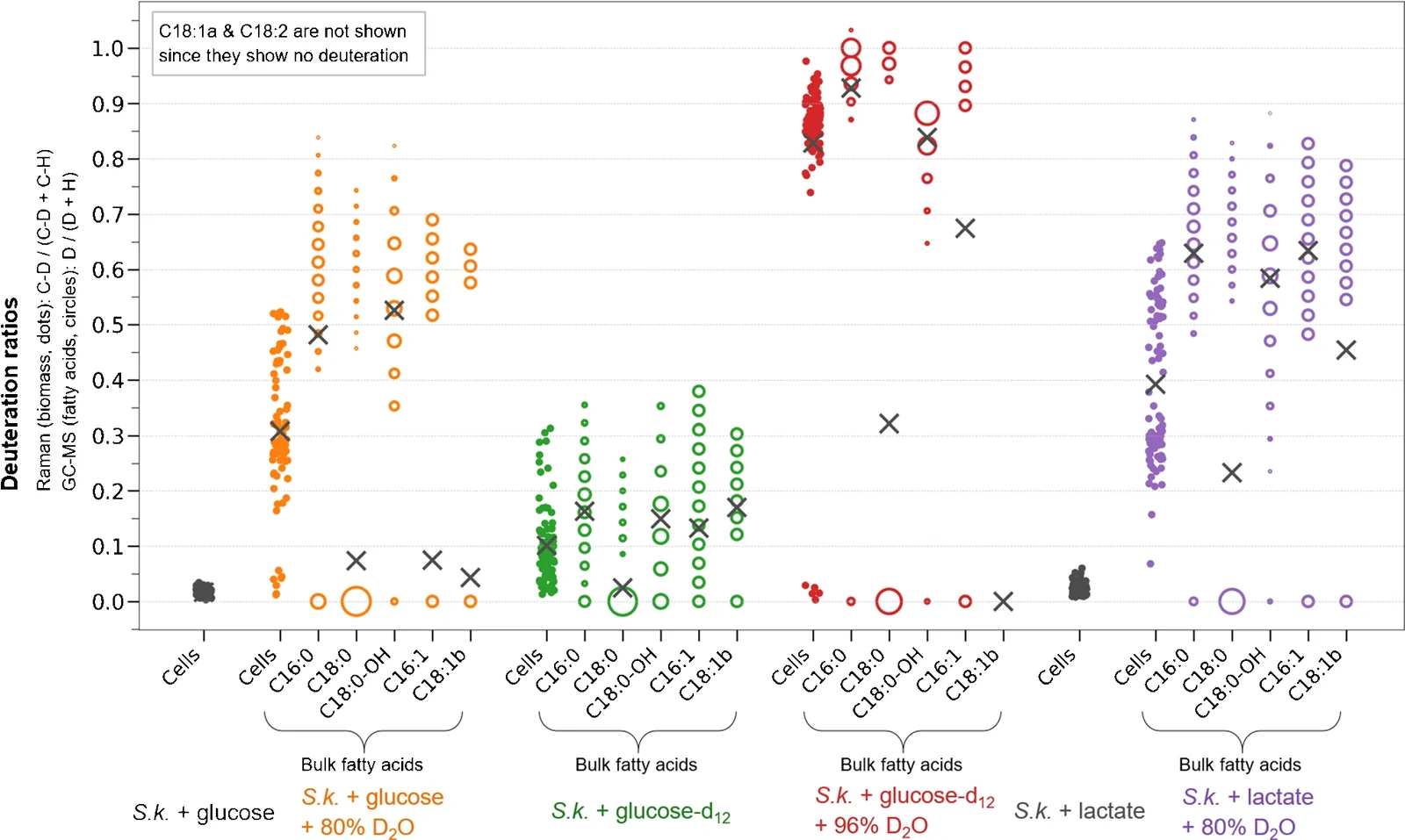
Comparison of biomass deuteration degrees of single *S. koreensis* cells determined with SIRM with deuteration degrees of different FAME isotopologues of entire populations calculated from mass spectra. The sizes of the circles of the mass spectrometry data are based on the integral size. All isotopologues with ion counts below the limit of quantification are depicted in the same size. The crosses indicate the mean deuteration of each FA. Attention has to be paid to the interpretation of the D degree of C$_{18:0}$-OH, since it has been calculated from the [M-113]$^{+}$ ion, which is a much smaller fragment and could thus lead to a greater uncertainty

While the deconvolution process of the D-isotopologues is straightforward for methyl esters of saturated fatty acids with only the [M]$^{+}$ and [M+1]$^{+}$ ions, it gets more complicated for unsaturated FAs, where more fragmentation ions are considered (see Fig. 1) and intensities are up to ten times smaller. Nevertheless, we achieved a similarly meaningful deconvolution of the D-isotopologue distribution of C$_{16:1}$ in the sample where *S. koreensis* was incubated with lactate and D$_{2}$O (sample with highest signal-to-noise ratio; see Supporting Information (SI) Figs. S2 + S3). Our fit indicates a binomial distribution with *n* = 29 H atoms and a deuteration probability of 65%. This deuteration probability is very close to the 68% calculated for C$_{16:0}$ in the same sample. The difference equals two D atoms and could hypothetically be explained by the desaturation process. However, biological triplicates with smaller standard deviations would be required to draw such further conclusions.

In comparison, for other FAMEs, the signal-to-noise ratios were too low to perform a similarly reliable deconvolution. We, therefore, relied on a simplified approach to describe the deuteration degrees. Based on the observation of Fig. 3f that deconvoluted D-isotopologue distributions showed strong agreement with binomial distributions, we resorted to using the median D-isotopologue to estimate FA deuteration degrees. For the C$_{16:0}$ example in Fig. 3d, the m/z with the strongest intensity (= the mode of the distribution) is 289. The non-deuterated FAME had a mass of 270, which means that 19 out of 31 hydrogen atoms in the most abundant peak were deuterated, which equals a deuteration degree of 61%. As shown in Fig. 3f, the simplified estimation of the deuteration degree from the mode (61%) is very close to the deuteration degree calculated from the binomial D-isotopologue distribution (62%). The simplified model also gave good agreement for unsaturated FAs (see C$_{16:1}$) (SI, Fig. S4, shows the simplified D degrees of all samples). Therefore, we bring forward this simplified approach as a convenient approach provided that binomial distributions prevail also in future studies.

### Complementary µ-Raman and GC-MS analyses

In Fig. 4, we summarize results from Raman (i.e., SIRM) and GC-MS (FAME) data. The samples grown on different substrates and with different D sources are indicated by different colors. Biomass deuteration degrees of cells incubated without a D source as determined by SIRM are given in grey (*S.k.*+glucose and *S.k.*+lactate). For all other samples, SIRM data are presented alongside the corresponding FAME-GC-MS data of different fatty acids. The first category of each sample on the *x*-axis, annotated as “Cells,” represents the single-cell SIRM data and the following five categories are named by the FAs (C$_{16:0}$, C$_{18:0}$, C$_{18:0}$-OH, C$_{16:1}$, and C$_{18:1b}$), which were characterized as methyl ester derivatives with GC-MS for bulk samples of microbial cells. The SIRM data points are all of the same size, as each represents the average deuteration degree of one single cell. Different non-deuterated, partially deuterated, and perdeuterated biomolecules (e.g., fatty acids, proteins, and DNA) can all contribute to the deuteration degrees measured by SIRM. The corresponding FAME-GC-MS data are presented as circles of different sizes, representing the relative abundances of the different D-isotopologues of each deuterated FA, as derived in Fig. 3. For FAME peaks with too low intensities (C$_{16:1}$ and C$_{18:1b}$), their D-isotopologue intensities could not be reliably calculated. Therefore, the masses were only used to determine the deuteration degree of each isotopologue, and all circles are shown in the same size (yes/no information), although binomial distributions can be expected. We note that the relative abundances (circle sizes) should only be compared within the isotopologue distribution of a given fatty acid and not between different FAMEs. The reason is that we did not calibrate to correct for different FAME-specific GC-MS response factors to account for effects of ionization efficiency, fragment intensity, and instrument performance of the day.

### Raman and GC-MS analyses validate each other’s results

Figure 4 shows that both methods can characterize deuterium incorporation with remarkable agreement between the incorporation of label into cells and into FA isotopologues for all samples. The highest degrees of deuteration were observed for the cells grown on glucose-d$_{12}$ in the presence of D$_{2}$O and the smallest for the ones grown on glucose-d$_{12}$ with both methods. For the cells grown on non-labelled glucose in the presence of D$_{2}$O, the cell and FA deuteration degrees are slightly lower than for cells grown on lactate in the presence of D$_{2}$O.

Both the SIRM and FAME-GC-MS data exhibit bimodal patterns, with values accumulating at zero deuteration and again at higher deuteration degrees, separated by a gap between these ranges. The upper cluster displays an internal structure, potentially comprising sub-clusters, as discussed in more detail later. Deuterium incorporation can occur during the de novo synthesis of new biomolecules or during metabolic processes that do not result in microbial growth (e.g., glycolysis followed by fatty acid synthesis). Hence, we conclude that non-labelled cells would neither be growing nor be metabolically active. Vice versa, deuterated cells can be interpreted as either growing or metabolically active.

As expected, we observe a higher fraction of non-deuterated fatty acids than of non-deuterated cells (i.e., see the sample grown on lactate in the presence of D$_{2}$O in Fig. 4, purple), since non-deuterated biomolecules can be present in both non-deuterated and deuterated cells. For the same reason, the degree of deuteration is higher for deuterated fatty acids than for deuterated cells, which can consist of a mixture of deuterated and non-deuterated biomolecules (fatty acids, proteins, DNA, carbohydrates, etc.). Furthermore, this reasoning is reinforced by the observation that the mean values of the cell deuteration degrees, which are indicated as crosses within the Raman data points (see Fig. 4) lie within the mean deuteration degrees of the different fatty acids of each sample (also marked as crosses).

Consequently, the µ-Raman and GC-MS data support each other. However, Fig. 4 reveals that there are also subtle differences that contain additional information about the heterogeneity in the label on the single cell level as indicated by SIRM; a heterogeneity between FAs on the level of different molecular structures (most pronounced in C$_{18:1a}$ and C$_{18:2}$, which showed no deuteration at all, data not shown), and a heterogeneity from isotopologue to isotopologue (i.e., the spread of the isotopologue distribution), as indicated by FAME-GC-MS and discussed in more details in the following.

### Single-cell deuteration degrees unravel phenotypic heterogeneity and suggest different cell generations within each sample

The variability in single-cell biomass deuterations within each experiment indicates phenotypic heterogeneity of *S. koreensis* cells with respect to metabolic activity. While being aware of the fact that single-cell deuteration degrees depend on both the cell’s growth state and its metabolic activity, here, we tentatively based calculations on the hypothesis that cell clusters with different mean deuteration degrees would primarily be indicative of different cell generations. With optical density as an independent, complementary indicator of cell growth, we observed an increase in OD600 of + 433% in the sample grown on glucose in the presence of 80% D$_{2}$O, where + 300% would represent the third and + 700% the fourth generation (see SI, Fig. S5). We assumed that in this sample, most label incorporation happened in the context of bacterial growth and D incorporation via other metabolic activities could be neglected.

Figure 4 shows three cell clusters for the cells grown on glucose in the presence of 80% D$_{2}$O (orange data points): one represents non-deuterated cells, the second shows cell deuterations of around 30%, and the third around 45%. The fraction of deuterated fatty acids (orange circles) shows deuterations of approximately 60%. If we consider binary cell division, a bacterial cell must double its biomolecules to achieve two equivalent cells. Assuming that all de novo synthesized biomolecules have similar deuteration degrees to FAs (around 60%) and that the cell consists of 50% initial non-deuterated biomass before division and of 50% de novo synthesized, deuterated biomolecules, we can expect the second generation cells to have biomass deuterations of 30%. If those cells divide again, third-generation cells should have biomass deuterations of (30%+60%)/2 = 45%. These two values coincide with the two clusters and support our hypothesis of different cell generations.

Similarly, the sample of *S. koreensis* cells incubated with lactate and 80% D$_{2}$O (purple data points), also shows two clusters, but with higher deuteration degrees than observed for the glucose sample. This is in accordance with the elevated fatty acid deuteration degrees of this sample.

Interestingly, no different clusters, indicating different generations, can be discerned for the population grown on glucose-d$_{12}$ in the presence of D$_{2}$O. Although this experiment exhibited the slowest cell growth (see OD600 values in SI, Fig. S5), it resulted in the highest levels of cell and FAME deuteration (see Fig. 4). In light of the ambiguity surrounding this observation, we advance two possible hypotheses that may elucidate the underlying phenomenon. (a) The cells need to adapt to the high D content within the medium. Once adapted, the cells grow continuously and are all within the same generation, leading to only one cluster of deuterated cells. (b) The cells are initially not able to grow in the highly deuterated medium, but are still metabolically active. Due to the high D content within the medium, these cells are already strongly labelled. After adapting to the new growing conditions, the already labelled cells replicate, causing the deuteration degrees of the different generations to shift to higher values and to overlap. This would also account for the high fraction of deuterated cells despite the slow cell growth.

### The degree of fatty acid deuteration varies statistically among different isotopologues and differs according to molecular structure

Whereas cell deuteration degrees have been shown to likely reflect the number of cell divisions, FA deuteration degrees are governed by biosynthetic incorporation processes rather than growth dynamics. Specifically, they depend on how the D source is incorporated during fatty acid biosynthesis and on the associated kinetic isotope effects. Since we observe isotopologues with different deuteration degrees for each fatty acid in a sample, and since these distributions follow binomial behavior, we can assume that each potential D-incorporation event occurs independently with a certain probability. The D-isotopologue distributions are narrowest for the fatty acids extracted from the cells incubated with glucose-d$_{12}$ and 96% D$_{2}$O because their deuteration levels approach the upper bound (100%), which inherently limits the possible spread of the data.

**Fig. 5:**
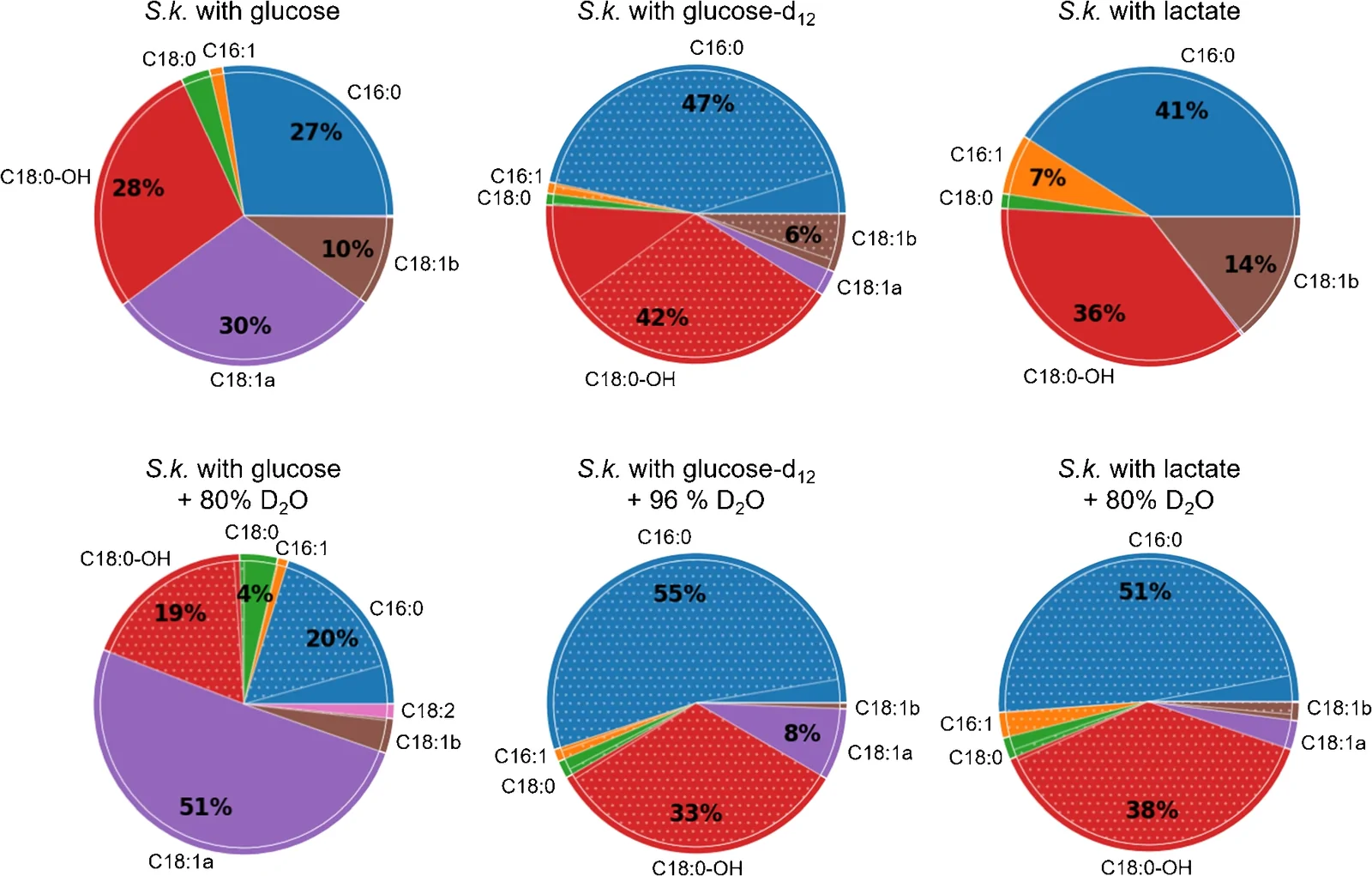
The pie charts indicate changing fatty acid profiles of *S. koreensis* when incubated with different substrates. The percentages represent the abundances of the sum of deuterated (dotted areas) and non-deuterated (plain areas) isotopologues for each fatty acid across all fatty acids within each sample and are not validated against a standard. Therefore, the actual fatty acid profiles may vary, and the charts are intended only to compare changes, i.e., a higher ratio of C$_{18:1a}$ in the samples with glucose and glucose + 80% D$_{2}$O. The mass traces of the following ions were chosen to describe the corresponding fatty acids: [M]$^{+}$ for C$_{16:0}$ (blue) and C$_{18:0}$ (green); [M-31]$^{+}$, [M-32]$^{+}$ and [M-33]$^{+}$ for C$_{16:1}$ (orange), C$_{18:1a}$ (purple), C$_{18:1b}$ (brown), and C$_{18:2}$ (pink); [M-113]$^{+}$ for C$_{18:0}$-OH

C$_{16:0}$ is generally known as the central FA, serving as a basis for the biosynthesis of most other FAs. Therefore, similar D degrees can be expected for FAs formed from C$_{16:0}$ via chain elongation, desaturation, or oxidation. This can be seen in Fig. 4 and SI, Fig. S4, where C$_{16:0}$, C$_{16:1}$, C$_{18:0}$, and C$_{18:1b}$ show only little variations in deuteration within each sample. Based on the differences, it would be possible to calculate how many D or H atoms are removed during desaturation reactions. However, we abstain from such interpretations due to the low signal-to-noise ratios for these compounds in our experiments. While de novo synthesized fatty acids showed similar deuteration degrees to C$_{16:0}$, we note that C$_{18:1a}$ and C$_{18:2}$ are exceptions which show no deuteration in all samples and, hence, are not de novo synthesized in our experiments.

### The deuterium source and carbon substrate influence deuteration levels

Beyond the observed variations in cell and FAME deuteration within the samples, deuteration levels are further modulated by the type of deuterium source and carbon substrate. To discuss how the deuterium source shapes FA deuteration degrees, we focus on C$_{16:0}$, which is a central FA and indicative of D assimilation because of its consistently high D fraction across all samples (see Fig. 5). During FA de novo synthesis, protons are obtained from water, the carbon source, and other metabolites. Consequently, the probability of FA deuteration is a function of the ratio of D$_{2}$O in the medium, and the deuterium content in the carbon source and in other metabolites.

In our experiments, we used D$_{2}$O, glucose-d$_{12}$, and a mixture of both to determine their effects on FA deuteration degrees during FA synthesis (see Fig. 4). However, we did not measure biological replicates, so it is not possible to state uncertainties. *S. koreensis* grown on glucose obtains approximately 78% of the hydrogen atoms for fatty acid biosynthesis from the medium in the form of water. This estimate is based on a mean D degree of C$_{16:0}$ of 62% in the sample grown on glucose in the presence of 80% D$_{2}$O (binomial distribution of orange data; 62% mean D degree of deuterated C$_{16:0}$/80% D in medium = 78%). Additionally, 18% of hydrogen in fatty acids is derived from glucose, as determined from the FAME deuteration of the incubation with glucose-d$_{12}$ (green data in Fig. 4). Together, water and glucose therefore account for 96% of the hydrogen used in fatty acid synthesis. The remaining 4% could originate from other metabolites (such as NADPH), which were formed before D was introduced, or from feeding of dead and therefore non-labelled cells. Moreover, the kinetic isotope effect could lower the D uptake from water, so that we would underestimate the contribution of water-derived hydrogen. Nonetheless, from these calculations, we can estimate an expectation value for the deuteration probability of the cells grown on glucose-d$_{12}$ and 96% D$_{2}$O and compare it with the observed value (Fig. 4, red data) to derive an order of magnitude of our error. The expectation value would be 93% for C$_{16:0}$ (78% H atoms from water × 96% D in medium + 18 % H atoms from C source × 100% D in glucose), which compares to an observed deuteration probability of 98% (Fig. 4, red data). Considering that we are comparing values achieved from populations grown in three different environments with different growth rates, those two values are in very good agreement.

Furthermore, the carbon substrate seems to influence FA deuteration degrees and the fraction of active, deuterated cells. This could be based on different active metabolic pathways. In this manner, we observe slightly higher FA and cell deuteration degrees for cells grown on lactate, compared to glucose in the presence of 80% D$_{2}$O (see Fig. 4). Moreover, similar cell deuteration fractions have been observed for the populations grown on glucose (glucose + D$_{2}$O, 40 out of 47 measured cells are deuterated = 85%; glucose-d$_{12}$, 62/75 = 83%, determined visually from single-cell spectra; glucose-d$_{12}$ + D$_{2}$O, 83/101 = 82%), while they differ significantly for the population grown on lactate as another carbon source (lactate + D$_{2}$O, 90/91 = 99%).

### Growth conditions modulate fatty acid profiles

The value of our analytical approach lies not only in analyzing heterogeneities in cell and fatty acid deuteration, but also in the outcome derived from the varying fatty acid compositions across samples, as outlined below. Different fatty acid abundances were observed depending on the deuterium source (D$_{2}$O and glucose-d$_{12}$)and carbon substrate. Since no calibration was attempted, we note that quantitative comparisons can only be made for the same fatty acid between experiments representing different growth conditions, and not for different fatty acids in the same experiment. Still, the data of Fig. 5 illustrate that fatty acids were produced or accumulated to different extents, resulting in changes also in the relative proportions of saturated and unsaturated fatty acids. Bacteria are known to regulate fatty acid biosynthesis to adapt membrane properties like chemical stability, stiffness, and permeability or to store saturated fatty acids as an energy source. Hence, the analysis of varying fatty acid profiles and their deuteration degrees holds great promise for analyzing such contributions in future studies including biological replicates. It can further serve as a guide to find suitable biomarkers for future studies. For example, C$_{18:0}$-OH showed consistently high deuteration fractions, especially for experiments where D$_{2}$O was used as a D source.

### The combined Raman and GC-MS approach can help to interpret Raman bands based on fatty acid deuteration

Based on Fig. 4, we showed how the single-cell Raman and the bulk fatty acid data nicely align and complement each other. Here, we take one step further and explore if FAME-GC-MS measurements can help to constrain a hypothesized assignment of a Raman C-D stretching band in a previous publication based on FA deuteration degrees. In a study where *S. koreensis* was grown on deuterated polylactic acid (micro)plastic particles [76], an additional C-D Raman stretching band at 2068 cm$^{-1}$, alongside the commonly discussed deuterated lipid, protein, and DNA bands, was highlighted. It was hypothesized that this additional band corresponded to C-D vibrations of strongly deuterated lipids (e.g., terminal CD$_{3}$). Here, we therefore explored the opportunity of fatty acid analysis by GC-MS, in addition to SIRM, for providing deeper insights into the discussion of this Raman band.

We only detected the Raman band at 2068 cm$^{-1}$ in the samples with average FA deuterations of at least 60% (see Fig. 2b). (For example, C$_{16:0}$ in the sample of *S. koreensis* with lactate and D$_{2}$O was 68 % deuterated.) Assuming that exactly 21 out of 31 hydrogen positions in the molecule were randomly replaced by deuterium (the D degree is 68 %), the probability that all three hydrogen atoms of the terminal methyl group were deuterated is 30% as given by the hypergeometric distribution (“sampling without replacement”). In contrast, for the sample with glucose-d$_{12}$ with a C$_{16:0}$ deuteration degree of 18%, the probability for a CD$_{3}$ group is only 0.4%. Therefore, if the 2068 cm$^{-1}$ Raman band represents strongly Raman active C-D stretching vibrations of a terminal CD$_{3}$ group in lipids, this would explain why it is not present for the sample with glucose-d$_{12}$.

In addition, due to its higher symmetry and polarizability compared to CD$_{2}$ groups, the C-D stretching vibration of the CD$_{3}$ group should have a higher Raman activity. Indeed, we observed a higher intensity of the 2068 cm$^{-1}$ Raman band than expected from the ratio of C-D bonds in CD$_{3}$ and CD$_{2}$ groups within lipids (i.e., 3/28=11% for C$_{16:0}$). CD$_{3}$ groups in proteins are closer to other electron-pulling groups than the CD$_{3}$ group at the end of an aliphatic chain in lipids. Due to the lower electron density at the protein CD$_{3}$ carbon, which results in a stronger C-D bond, we can distinguish C-D stretching vibrations of methyl groups in lipids and proteins based on their Raman frequencies, with the protein signals appearing at higher wavenumbers. Therefore, the analysis of FA deuteration patterns supports our hypothesis that the 2068 cm$^{-1}$ Raman band originated from strongly deuterated lipids, which have a terminal CD$_{3}$ group.

## Conclusion

In this study, we bring forward evidence that the proportion of deuterated cells and cell deuteration degrees obtained with SIRM (single-cell-SIP approach) are in good agreement with the fraction of deuterated FAMEs and FAME deuteration degrees derived from FAME-GC-MS data (compound-specific SIP approach). This consistency was observed when cells of the same microorganism were grown in the presence of substrates and deuterium label in different forms (glucose, lactate, D$_{2}$O, and glucose-d$_{12}$).

Hence, the combination of both SIP approaches extends the capabilities of each individual method by enabling a multiscale understanding, encompassing single-cell and bulk-level analyses, and whole-cell profiling and the targeted characterization of specific metabolites. While the single-cell SIRM information unraveled phenotypic heterogeneities in metabolic activity and growth, the FAME-GC-MS bulk analysis ensured the representability of the single-cell data. The analysis of fatty acids as specific metabolites demonstrated heterogeneities in the labelling of different fatty acids, their isotopologues, and in fatty acid profiles under different isotopic compositions or substrates, reflecting changes in bacterial physiology by environmental factors. The whole-cell SIRM data, in contrast, put the single metabolite data into the frame of entire organisms and indicated the presence of different cell generations. In summary, we showed that SIRM and FAME-GC-MS data not only validate, but also complement each other.

With this foundation—the demonstrated value of a combined compound-specific- and single-cell SIP methodology in the mono-species experiments of this proof-of-principle study—the same approach can be extended to more complex systems. The overarching aim of SIP methods is to link functional activity with microbial identity in natural systems, as summarized by Dumont and Murrell [1] and by Kreuzer-Martin [2]. The next step is therefore to work with microbial communities. In such an approach, an additional strength of the SIRM and FAME-GC-MS combination could be explored: the high lateral resolution and confocality of SIRM enable spatial mapping to discover microenvironments within complex samples, and FAME-GC-MS can additionally provide taxonomic information. A promising workflow would be to target active cells, to select them by Raman cell sorting based on their D incorporation, and to analyze the fatty acid composition and labeling of different cell fractions with FAME-GC-MS while gaining taxonomic details.

Possible applications could extend to investigating the biodegradation of deuterated (micro)plastics in natural systems. The D label incorporation from deuterated polylactic acid into microbial biomass was previously investigated using SIRM in mono-species as well as in soil community experiments [76] and would benefit from such a synergistic SIRM + FAME-GC-MS approach. In summary, our work paves the way for linking bacterial function to bacterial physiology and, potentially, taxonomic identity and spatial resolution in future research.

## Supplementary Information

Below is the link to the electronic supplementary material.

Supplementary file 1 (pdf 443 KB)

## Data Availability

Data that support the findings of this study are available from the authors on request.
