# Supplementary material for: µ-Raman and GC-MS to interrogate the heterogeneity of deuterium-label incorporation on the single cell vs. fatty acid isotopologue level
Source: Anal Bioanal Chem. 2026 Apr 29;418(23):7953–68. doi: 10.1007/s00216-026-06452-y (PMC13744501; doi:10.1007/s00216-026-06452-y)
Supplement: Supplementary file 1 [file 216_2026_6452_MOESM1_ESM.pdf]

Supporting Information

µ-Raman and GC-MS to Interrogate the Heterogeneity of Deuterium-Label Incorporation on the Single Cell vs. Fatty Acid Isotopologue Level

Kara Müller[1], Natalia P. Ivleva[1*], Claudia Huber[2], Wolfgang Eisenreich[2], Martin Elsner[1*]

*Corresponding authors. E-mails: natalia.ivleva@tum.de; m.elsner@tum.de
Contributing authors: kara.mueller@tum.de; claudia.huber@tum.de; wolfgang.eisenreich@mytum.de

[1]Institute of Water Chemistry, Chair of Analytical Chemistry and Water Chemistry, TUM School of Natural Sciences, Technical University of Munich, Lichtenbergstraße 4, Garching, 85748, Germany.
[2]Structural Membrane Biochemistry, Bavarian NMR Center, TUM School of Natural Sciences, Technical University of Munich, Lichtenbergstraße 4, Garching, 85748, Germany.

1. Detailed Stable Isotope Raman Microspectroscopy Data Analysis Procedures

Pre-processing of single-cell Raman spectra. First, the wavenumbers were corrected based on the Si Raman band at 520 cm^{-1} of daily Si-wafer measurements. Then all spectra were smoothed using a Savitzky-Golay-filter with a window length of seven and a polynomial order of three, using the Savitzky-Golay-function of the scipy.signal package. Next, the baseline of single-cell spectra was corrected with a statistics-sensitive non-linear iterative peak-clipping (SNIP) algorithm from the pybaselines.smooth package and additionally the mean intensity of the Raman-silent regions 1850 - 2050 cm^{-1} and 2350 - 2600 cm^{-1} was subtracted. Finally, the spectra were normalized to the integral of the C-D (1960 - 2400 cm^{-1}) and C-H (2800 - 3030 cm^{-1}) stretching vibrations.

Calculation of C-D and C-H stretching vibration band areas of single-cell Raman spectra.

The scipy.optimize.curve_fit function was applied, using non-linear least squares, to fit the sum of five gaussian functions to the C-D (1960 - 2400 cm^{-1}) and C-H (2800 - 3030 cm^{-1}) stretching vibration regions each. The parameters used to find the optimized parameters for the five gaussian functions of both wavenumber regions are summarized in Table S1. The optimized parameters of the Gaussians were then used to calculate their integrals with the composite trapezoidal rule by applying the scipy.integrate.trapz function.

Table S1: The following fitting parameters (intital guess, lower boundary, and upper boundary) of each Gaussian parameter were used to describe the C-D and C-H strecthing vibrations of the single-cell Raman spectra.

parameter	C-D vibrations	C-H vibrations
wavenumber region	1950 - 2400 cm^{-1}	2750 - 3030 cm^{-1}
initial guess of amplitudes / a.u.	0.00006, 0.00008, 0.00006, 0.00004, 0.00004	0.0009, 0.005,0.005, 0.005, 0.003
lower boundaries of amplitudes / a.u.	always 0	always 0
upper boundaries of amplitudes / a.u.	always 0.1	always 0.1
initial guess of center of the peaks / cm^{-1}	2070, 2113, 2148, 2196, 2223	2848, 2880, 2910, 2932, 2970
lower boundaries of center of the peaks / cm^{-1}	2060, 2103, 2138, 2186, 2213	2840, 2861, 2895, 2921, 2955
upper boundaries of center of the peaks / cm^{-1}	2080, 2123, 2158, 2206, 2233	2860, 2890, 2920, 2938, 2980
initial guess of standard deviations / cm^{-1}	always 15	always 15
lower boundaries of standard deviations / cm^{-1}	always 1	always 1
upper boundaries of standard deviations / cm^{-1}	always 70	always 50
initial guess of y_0 offsets / a.u.	always 0	always 0
lower boundaries of y_0 offsets / a.u.	always 0	always 0
upper boundaries of y_0 offsets / a.u.	always 0.001	always 0.001

2. Detailed Fatty Acid Methyl Ester - Gas Chromatography - Mass Spectrometry Data Analysis Procedures

2.1 GC-MS data import into python.

The .ms data files obtained from the Agilent GC-MS system were first converted to .mzML files using software OpenChrome (Lablicate, Germany) and then to .txt files using the MSExportGUI (Adusumilli & Mallick, 2017) of ProteoWizard.

2.2 Data analysis steps to calculate the integrals of single ion mass traces with the aim to determine FAME deuteration fractions and simplified FAME deuteration degrees.

1. The masses were rounded to integers to simplify the subsequent code. The molar mass of the $C_{16:0}$ FAME is 270.4558 g/mol, while that of the perdeuterated molecule is 301.6449 g/mol. Accordingly, values below x.7 were rounded down to the nearest lower integer, whereas values of x.7 and above were rounded up to the nearest higher integer.

2. All GC-MS data is stored in one pandas dataframe, where the columns represent the mass traces m/z = 200-350 plus one column with the time values, and the rows represent the different samples, separated by trpilicates (e.g., *S. koreensis* incubated with glucose, trpilicate i). The dataframe is filled with lists, all of the same length and saved in a pickle cache, to save computation time when changes are applied to the code.

3. For each FA, a dictionary for the non-deuterated isotopologue was manually created, which includes the following parameters to calculate the trapezoidal

integrals with values for each sample: m/z of single mass traces of interest (e.g. 270 and 271 for $C_{16:0}$ and 236, 237 and 238 for $C_{16:1}$), x_{min}, x_{max}, y_{max} and y_{min}. It further includes parameters for the corresponding plots.

4. The integrals of each non-deuterated fatty acid peak of each samples' triplicate is calculated and the chromatogram snippets are plotted for each triplicate and each fatty acid, separately with colored peak areas as a control mechanism to check whether the right area was chosen.

5. The integrals of the chromatogram peaks of interest are stored in dictionaries with the m/z values as keys and peak areas as values for each fatty acid and sample. Those dictionaries are then collected in a dataframe, which has the fatty acids as columns and the samples's triplicates as indices, and cached with pickle. The procedure is repeated for each sample until the entire dataframe in the cache is filled.

A similar pickle cache is created for the deuterated isotopologues:

6. For each FA, a function is manually prepared, including all necessary parameters in form of a dictionary. The samples are the keys and a nested list of all parameters is appended as values:
starting with a list of the measured m/z values in the chromatogram peak of the deuterated FA, followed by x_{min}, x_{max} for the calculation of the integrals and for the calculation of the plots,
y_{min} and y_{max} for the plot and the m/z range that should be shown in the plot. (Not all mass traces are always shown to avoid overlap of different ions, especially $[M]^+$ with $[M-31]^+$ and so on.)

7. The peak areas are again calculated as trapezoidal integrals and stored by extending the dictionaries in the dataframe within the cache of non-deuterated isotopologues, so that all data is collected in one big dataframe.

The sum of all integrals per fatty acid were used for the fatty acid profile pie charts in Figure 5 of the main manuscript. The integrals of the mass traces of non-deuterated and deuterated isotopologues are summed up separately to calculate the deuteration fractions of the fatty acids in the same plot. The same cache was further used to calculate the simplified deuteration degrees in the SI, Figure S4.

2.3 Deconvolution of D-isotopologues.

For the deconvolution of different D-isotopologues, as shown in Figure 3d of the main manuscript for a saturated FAME and in SI, Figure S2 for an unsaturated FAME, an additional script was written. In principle, we formulated two linear least-squares problems for each sample - fatty acid combination. The first was to calculate the deconvoluted integrals of the distributions of the non-deuterated isotopologue and the second for the deuterated isotopologues. Boundary conditions were set and the equations were solved using the scipy.optimize.lsq_linear function. The D-istopologue distributions were then visualized as in Figure 3e of the main manuscript for a saturated and in SI, Figure S3 for an unsaturated FAME, showing additional fits of binomial distributions.

The linear algebra problem used for the deconvolution of deuterated, unsaturated FAMEs can be described as:

$$\textbf{coefficient matrix (m x n)} * \textbf{isotopologue vector (length: n) =}$$
$$\textbf{intensity vector (length: m)}$$

The coefficient matrix (m x n) is constructed with the following assumptions:

1. We assume that the ratio of the $[M\text{-}32]^+$ / $[M\text{-}31]^+$ ions measured of non-deuterated FAMEs would be the same as the ratio of ($[M\text{-}32]^+$ + $[M\text{-}33]^+$) / $[M\text{-}31]^+$ ions of deuterated samples. For $C_{16:1}$ the ratio was determined as 1.5 and for $C_{18:1b}$ as 2.2.

2. The ratio of the isotopologues containing no ^{13}C (P_{12}) or one ^{13}C atom (P_{13}) can be calculated based on the BERNOULLI distribution and a natural ^{13}C abundance of 1.1% (see Table S1).

3. The deuteration degree of each D-isotopologue ($Deut_j$) is directly proportional to the intensity ratio of the [M-33] / ([M-32]+ [M-33]) fragments.

For different fatty acids, different coefficient matrices were compiled:

(a) <u>for deuterated, saturated fatty acids:</u>

We compiled a square matrix (m=n) and do not involve the mass of the heaviest D-isotopologue with one ^{13}C atom in the calculations, since this can be assumed to be very low, leading to a greater uncertainty. However, it is calculated in the end

Table S1: Probabilities of all ^{12}C-atoms or one ^{13}C-atom in the carbon chains of the analyzed FAMEs, based on the natural ^{13}C abundance of 1.1%. For the methyl esters of saturated and unsaturated C_{16} and C_{18} fatty acids, 17 and 19 C-atoms were considered, respectively, because of the additional methyl ester carbon. For $C_{18:0}$-OH the C_{11} fragment was used.

	sat./unsat. C_{16} FAs	sat./unsat. C_{18} FAs	C_{11} of $C_{18:0}$-OH
P_{12}: probability of only ^{12}C-atoms in carbon chain	82.9%	81.0%	88.5%
P_{13}: probability of one ^{13}C-atom in carbon chain	15.7%	17.1%	10.8%

from the natural ^{13}C abundance and considered for the intensity of the corresponding D-isotopologue. A (n x n) all-zero matrix is filled with the following matrix elements to describe the coefficient matrix with i as index for the rows and j as index for the columns:

- diagonal elements (i = j): coeff[i,j] = P_{12}
- first subdiagonal elements (j = i-1): coeff[i,j] = P_{13}

(b) for deuterated, unsaturated fatty acids:

For each D-isotopologue we get the four consecutive masses a-d, which describe the intensity of the following fragments (as schematically shown in Figure 1 of the main manuscript):

$$\begin{aligned} a:&\quad [M-33,\ ^{12}C]^+ \\ b:&\quad [M-33,\ ^{13}C]^+ + [M-32,\ ^{12}C]^+ \\ c:&\quad [M-32,\ ^{13}C]^+ + [M-31,\ ^{12}C]^+ \\ d:&\quad [M-31,\ ^{13}C]^+ \end{aligned}$$

, where ^{12}C stands for the isotopologue with only ^{12}C-atoms and ^{13}C for the isotopologue with one ^{13}C-atom.

These masses overlap and for n D-isotopologues, we detect n + 3 masses (see Figure 1 of the main manuscript). However, we do not consider the highest mass for the calculations again, since it only describes the [M-31, ^{13}C]$^+$ ion and its intensity is much lower than for the other masses where intensities of several ions and isotopologues are summed up. Therefore, we use a (n+2 x n) matrix. We formulated the linear algebra problem in dependency on the [M-31] fragments. The different fragments of one D-isotopologue (j) that encompass both isotopologues with and without

one ^{13}C atom can be formulated in dependency on the [M-31] fragment as follows:

$$\begin{aligned} M-31 &= X \\ M-32 &= \frac{ratio_{32/31}}{1+Deut_j} * X \\ M-33 &= \frac{Deut_j * ratio_{32/31}}{1+Deut_j} * X \end{aligned}$$

With these dependencies, we can compile the (n+2 x n) coefficient matrix by filling an all-zero matrix with the following matrix elements, where i is used as index for the rows and j as index for the columns (=different D-isotopologues):

- diagonal elements (j = i):
  coeff[i,j] = (Deut$_j$ * ratio$_{32/31}$) / (1 + Deut$_j$) * P$_{12}$
- first subdiagonal elements (j = i-1):
  coeff[i,j] = ratio$_{32/31}$ / (1 + Deut$_j$) * P$_{12}$ + (Deut$_j$ * ratio$_{32/31}$) / (1 + Deut$_j$) * P$_{13}$
- second subdiagonal elements (j = i-2):
  coeff[i,j] = P$_{12}$ + ratio$_{32/31}$ / (1 + Deut$_j$) * P$_{13}$
- third subdiagonal elements (j = i-3):
  coeff[i,j] = P$_{13}$

Isotopologue vector (length: n):
This vector is of length n and has to be solved in this linear algebra problem. Each value stands for the [M-31]$^+$ ion of one D-isotopologue, including the [M-31+1]$^+$ ion with one ^{13}C atom.

Intensity vector (length: m):
While this vector is of lengthn for saturated FAMEs, it is of length n+2 for unsaturated FAMEs, with n being the number of D-isotopologues. It includes the intensities of all masses of deuterated isotopologues that were detected for one fatty acid in one specific sample.

Exception: Low deuteration degree in experiment where *S. koreensis* is incubated with glucose-d$_{12}$
In the presence of the deuterated carbon source as only D source, very small deuteration degrees of the FAMEs were obtained, often starting with one D atom per molecule. This means that the mass of the ^{12}C-isotopologue of the first deuterated analog overlaps with the mass of the non-deuterated FAME with one ^{13}C atom. Therefore, the contribution of the ^{13}C-isotopologue of the non-deuterated isotopologue is first estimated based on the intensity of the non-deuterated ^{12}C-isotopologue and the natural

^{13}C abundance (see Table S1). The non-deuterated ^{13}C-isotopologue intensity is then subtracted from the intensity of the ^{12}C-isotopologue of the first D-isotopologue and the calculations are proceeded as previously discussed.

Exception: Full FAME isotopologue deuteration in experiment where *S. koreensis* is incubated with glucose-d$_{12}$ and D$_2$O

In case of perdeuteration, it is impossible to have the elimination of CH_3OH, leading to the [M-32]$^+$ ion. Therefore, its contribution is removed from the coefficient matrix.

3. Additional Figures

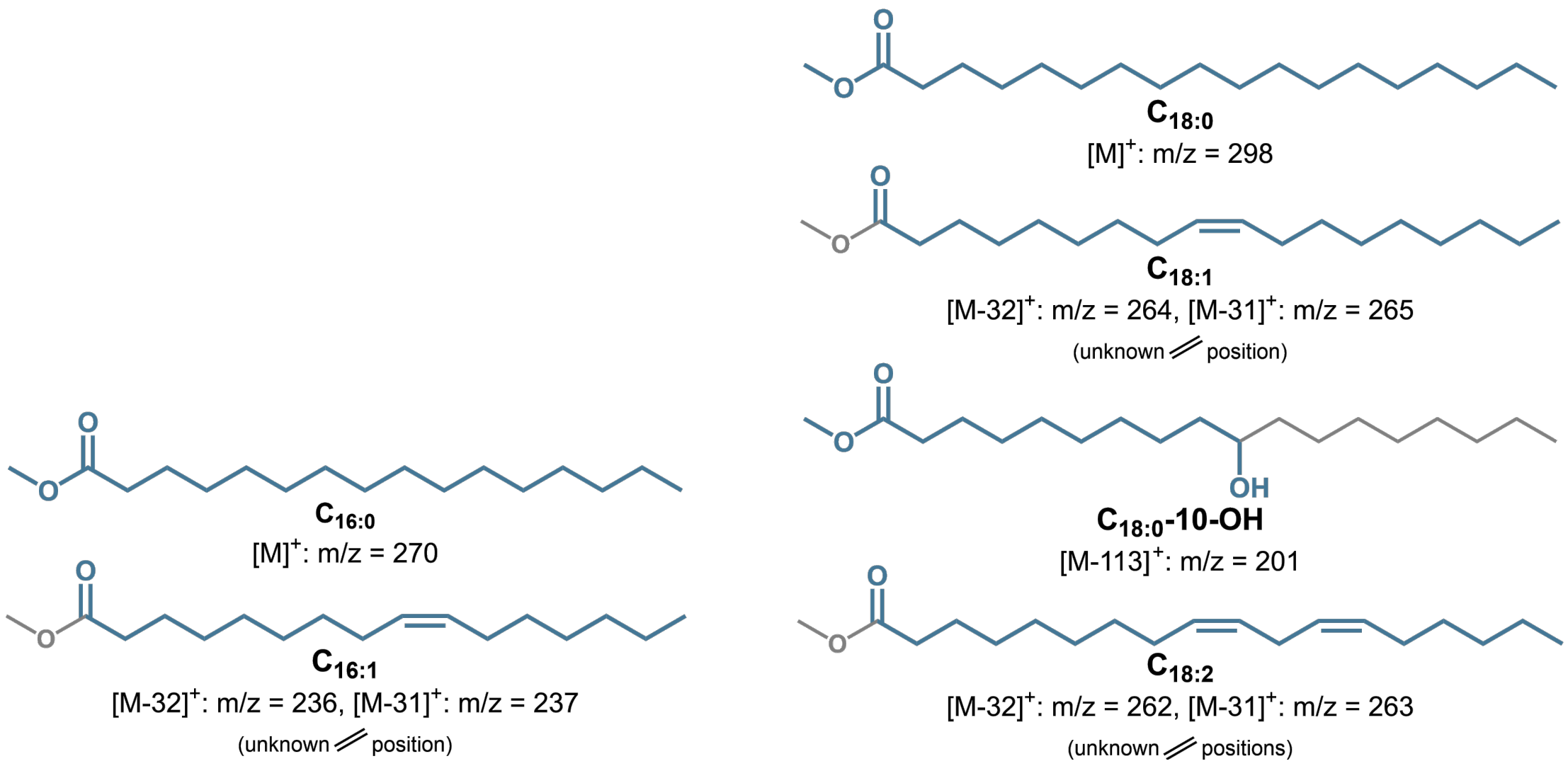

Figure S1: Structural formulas of a) C_{16} and b) C_{16} FAMEs, which were subject of this study. The ions used to identify these FAMEs in this study are highlighted (blue, bold) and their molecular weights are annotated. The position of the double bonds cannot be determined solely based on the mass spectra and is shown exemplarily.

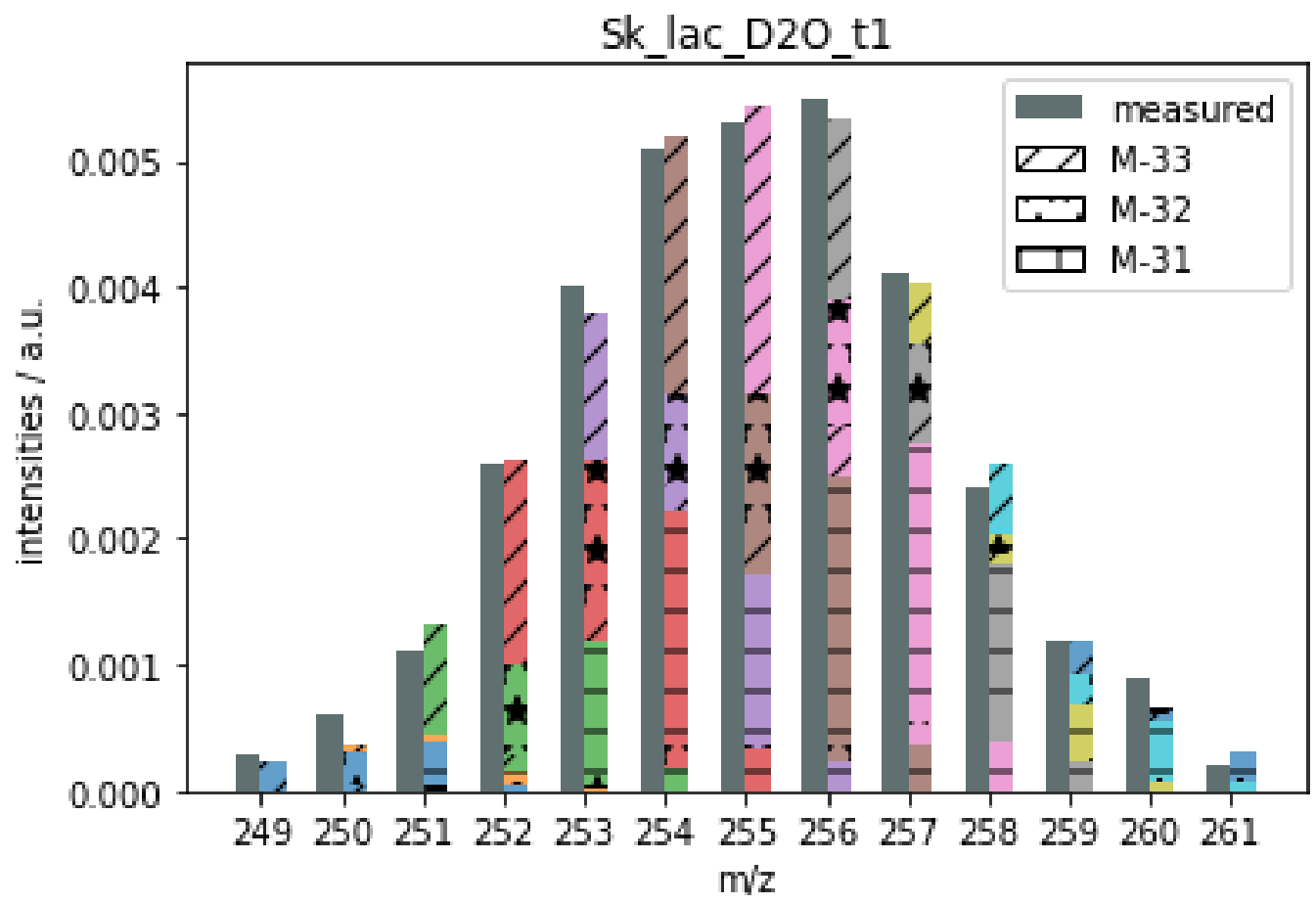

Figure S2: For deuterated, unsaturated FAME, the $[M\text{-}31]^{+}$ (horizontal lines), $[M\text{-}32]^{+}$ (stars) and $[M\text{-}33]^{+}$ ions (diagonal lines) overlap, with isotopologues of other deuteration degrees. The corresponding peaks and those of their isotopologues with one ^{13}C atom were deconvoluted for mass spectra with sufficient ion counts, as shown for this example.

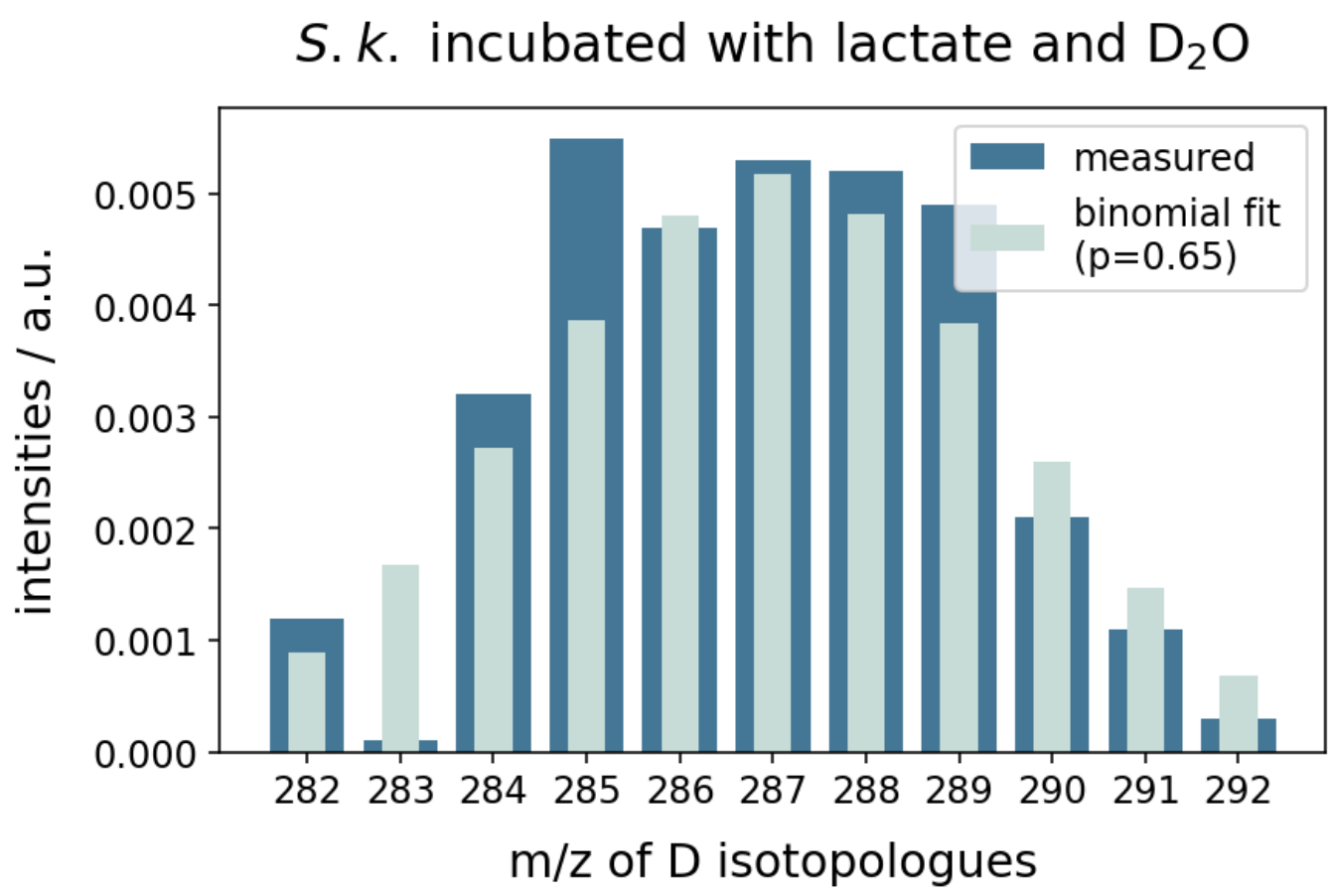

Figure S3: A binomial distribution was fit to the D-isotopologues distributions of $C_{16:1}$ for *S. koreensis* incubated with glucose-d_{12} and 96% D_2O to determine the deuteration degrees.

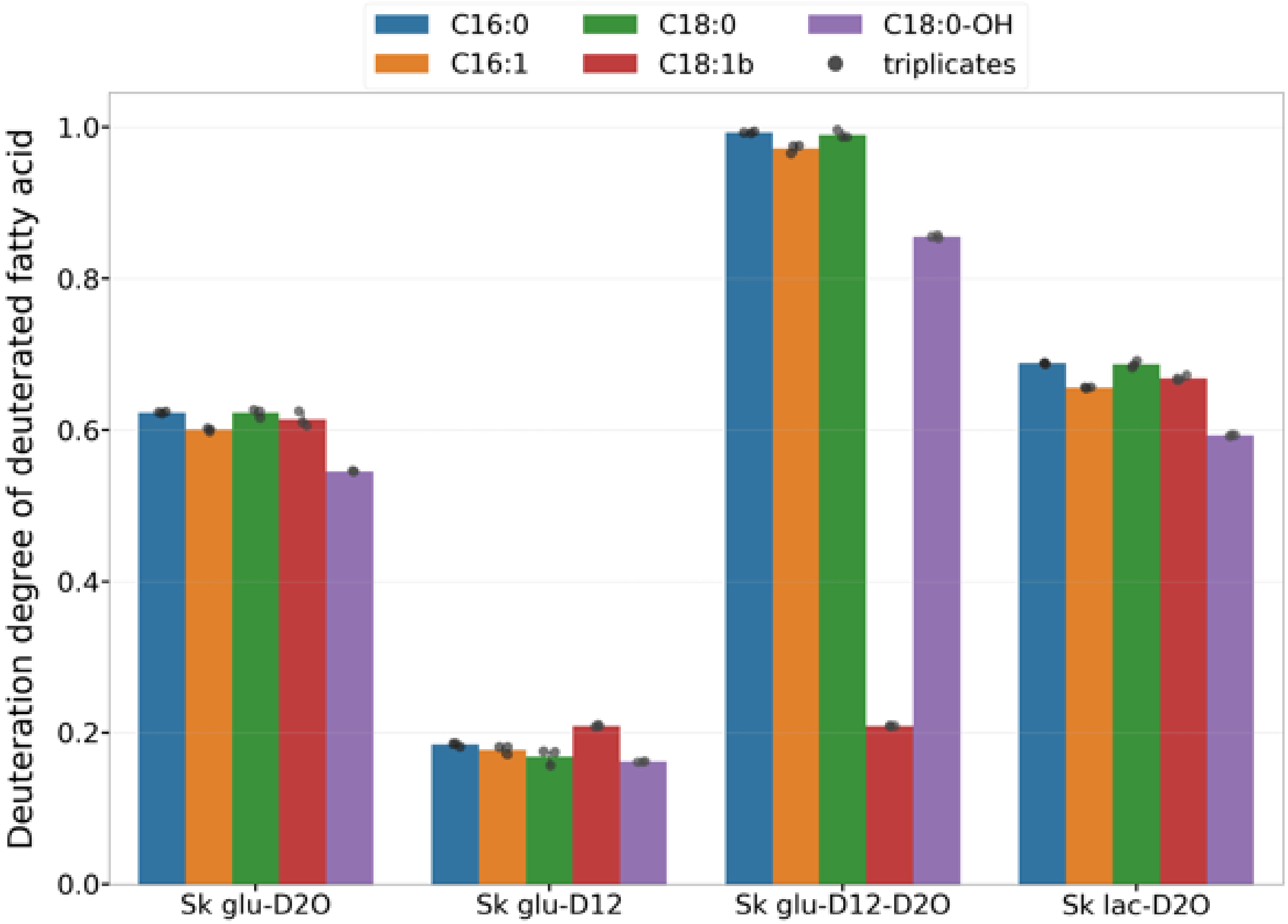

Figure S4: The FAME deuteration degrees are estimated from the most abundant m/z in the mass spectrum of the deuterated isotopologue and shown for all four experiments with deuterium. The single points show the variation of the technical triplicates.

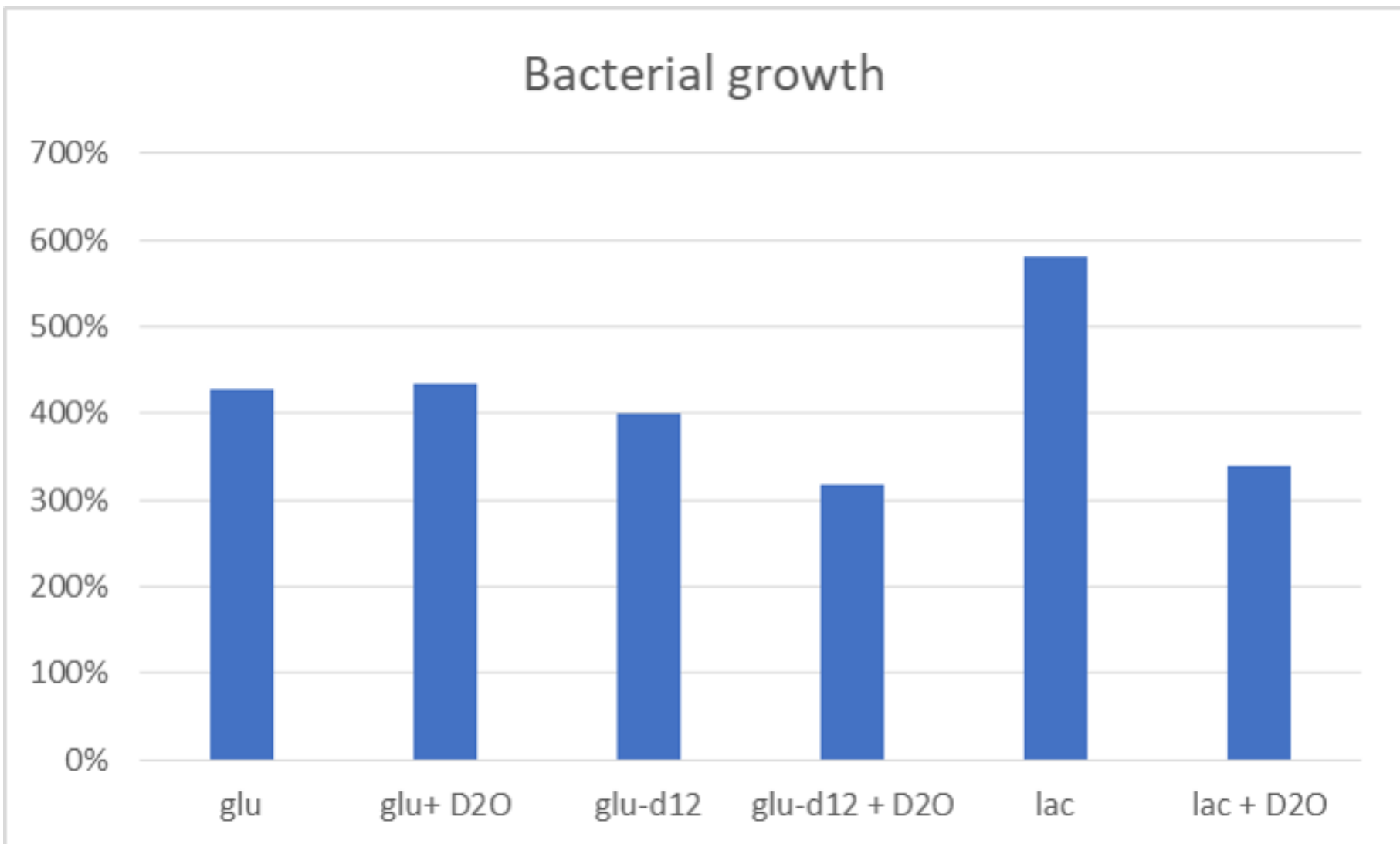

Figure S5: The bacterial growth of *S. korrensis* cells incubated with different substrates and D-sources was determined based on OD600 values of the initial cell suspensions (OD600$_{initial}$) and on the date of SIRM and FAME-GC-MS measurements (OD600$_{final}$). It is calculated as (OD600$_{final}$ - OD600$_{initial}$)/OD600$_{initial}$, so that a value of 100% corresponds to two times the initial cell density.

Bibliography

Adusumilli, R., & Mallick, P. (2017). Data conversion with proteowizard msconvert. In L. Comai, J. E. Katz, & P. Mallick (Eds.), *Proteomics: Methods and protocols* (pp. 339–368). Springer New York. https://doi.org/10.1007/978-1-4939-6747-6_23
